# Study of disbudding goat kids following injection of clove oil essence in horn bud region

**Published:** 2015-03-15

**Authors:** Mohammad Mahdi Molaei, Ali Mostafavi, Reza Kheirandish, Omid Azari, Mohsen Shaddel

**Affiliations:** 1 *Department of Clinical Sciences, Faculty of Veterinary Medicine, Shahid Bahonar University of Kerman, Kerman, Iran; *; 2 *Department of Chemistry, Faculty of Sciences, Shahid Bahonar University of Kerman, Kerman, Iran; *; 3 *Department of Pathobiology, Faculty of Veterinary Medicine, Shahid Bahonar University of Kerman, Kerman, Iran; *; 4 *Graduate student, Faculty of Veterinary Medicine, Shahid Bahonar University of Kerman, Kerman, Iran.*

**Keywords:** Clove essence, Disbudding, Goat kid

## Abstract

This study was performed to evaluate the efficacy of injection of essential oil of *Eugenia caryophyllata* in the kid horn buds, as a new chemical technique for disbudding. Five-day-old healthy goat kids from both sexes (n = 16) were divided randomly into 4 equal groups. In groups 1, 2 and 3, 0.2 mL of clove essence and in group 4 (control) 0.2 mL of normal saline was injected into the left horn bud of goat kids. Right horn bud in all kids was considered to ensure that they are horned. During the study, the rate of horn growth were evaluated in determined time intervals between groups 1 and 4. Tissue samples were taken from right and left horn bud in groups 2 and 3, at five and ten days after clove essence injection, for microscopic study. The results of the study showed that the clove essence stopped horn growth, whereas there was no significant difference in horn growth rate between left and right horns after injection of normal saline, in group 4. Histopathological study showed that injection of clove essence caused complete necrosis of epidermis and underlying dermis with collagenolysis in horn bud tissues, 5 days after injection and then progress in healing process was observed after 10 days. According to the results of this study, it can be concluded that the injection of clove essence is an effective method to stop horn growth without any undesirable effects on clinical parameters in goat kids.

## Introduction

Horns are the pairs of hard, bone like permanent growths projecting from the heads of certain mammals, such as goat, consisting of a bony core covered with a sheath of keratinous material.^1^ In the wild, horns play an important role as natural weapons that provide protection to animals against predators.^2^ Horns have no useful function in a domestic goat.^3^ Animals with horns are more apt to fight with one another or be aggressive toward people. The horned animals are more likely to destroy farm facilities and to be entrapped in fences.^4^ Horns occasionally grow in such a manner as to press on soft tissues or obscure vision.^3^ Some goat breeds cannot be registered or shown until they are dehorned.^4^

Nowadays depending on age, various techniques of dehorning have been described for small ruminants. These methods include chemical (caustic paste), thermal cautery (hot iron), cutting (sawing), rubber bands or genetic procedures. The risks to the goat and the operator vary with each technique.^5^ Reduction in milk production, impairment of spermatogenesis, sinusitis and myiasis and loss of social status in the herd are other side effects of dehorning in the mature goat.^6^^,^^7^

Disbudding is usually performed within the first week of life to remove horn buds in young goats.^4^^,^^8^ Chemical and thermal methods are commonly used for disbudding in goat kids. Both procedures will destroy the horn cells (disbudding) and prevent growth. However, these methods may have some complications such as injury to the surrounding tissues (skin, frontal bone, brain and eye).^9^ Disbudding by thermal cauterization induces an acute cortisol elevation and increases the expression of behaviors that indicate stress and pain. Goats appear to be extremely sensitive to pain and may be fractious when retrained.^10^

Eugenol (4-allyl-2-methoxyphenol) is the main component of oil of cloves *Eugenia caryophyllata *(syn. *Syzigium aromaticum*).^11^ Essential oil extracted from the cloves contains almost 72 to 90% eugenol. Cloves are widely grown in Indonesia, Madagascar and also in other countries like India and Sri Lanka. Eugenol is a natural phenolic compound is present in reasonable amounts in several other spices like basil, cinnamon and bay leaves. Eugenol has been used as a flavoring agent in cosmetics and food products and also plays a role in dentistry as cavity filling cement. Eugenol is said to possess various biological properties like antiviral, antioxidant, anti-inflammatory, *etc*. At low concentrations, it usually acts as an antioxidant and anti-inflammatory agent, whereas at higher concentration, act as a pro-oxidant causing increased generation of tissue-damaging free radicals. It has been reported to possess anti-genotoxic activity.^11^

Numerous studies have indicated that eugenol is cytotoxic to mouse fibroblast cell line L929,^12^ rat hepatocytes,^13^ pulp cells^12^^,^^14^ and oral mucosal fibroblasts^15^ and osteoblastic cells *in vitro*.^16^ Eugenol was also found to cause injury to rat oral mucosa membranes *in vivo*.^16^^,^^17^ It is stated that eugenol could decrease growth of various kind of neoplastic cells via induce of apoptosis and inhibition of cell proliferation.^18^^-^^20^

The purpose of this study was to determine the efficacy of injection of essential oil of *E. caryophyllata* (EC) in kid horn bud, as a new chemical technique for disbudding in goats.

## Materials and Methods

**Preparation of essential oil of** ***E. caryophyllata*** **. **Fresh spice (Clove) was bought from a main market in Kerman, Iran. They were milled to fine powder with electric blender. Two hundred gram of the powder mixed with 700 mL distilled water in a clevenger-type apparatus equipped with an electric mantel heater for 3.5 hr. The essential oil was extracted by traditional hydrodistillation method.^21^^,^^22^ After providing the essence, gas chromatography/mass spectrometry (GCMS) analysis (Model 17A-GC & QP5000-MS; Shimadzu Kyoto, Japan) was done to identify of essence component.

**Animals. **Five-day-old healthy Raeini goat kids (n =16) from both sexes were divided randomly into four equal groups. In groups 1, 2 and 3, 0.2 mL of clove essence and in group 4 (Control) 0.2 mL of normal saline was injected into the left horn bud of goat kids. Right horn bud in all kids was considered as a normal group, to ensure that they are horned. Groups 1 and 4 were considered for macroscopic study and groups 2 and 3 for microscopic study.

**Macroscopic study.** The speed of horn growth was evaluated by measuring of horn height (distance from base to tip of horn) with a fine caliper. The data of horn height was recorded during two months study and compared statistically, after that; the horn growth was evaluated clinically between the groups for a 12-month study period.

**Microscopic study.** Under local anesthesia with lidocaine hydrochloride 1%, tissue specimens (0.5 cm × 0.5 cm) were taken from right and left horn buds, 5 and 10 days after injection of clove essence in groups 2 and 3, respectively. The samples from the skin bud and underlying tissue were fixed in 10% buffered formalin. The samples were embedded in paraffin, sectioned at 4 μm, and stained with hematoxylin and eosin (H & E) for light microscopy.

**Statistical analysis.** Data analyses were performed using SPSS software (Version 16; SPSS Inc., Chicago, USA). The recorded data of the speed of horn growth were analyzed with repeated measurement analysis of variance for statistical comparison. Significance was set at *p* < 0.05. The histopathological findings were compared between right and left horns of animals in groups 2 and 3, descriptively.

## Results

The essential oils were found to be rich in eugenol compounds representing 85.04% eugenol, 12.02% aceto-eugenol, and 0.21% dehydrodieugenol of the total oil. The GCMS analysis of the clove essence was presented in Table 1.

**Table 1:** Component of clove essence identified by gas chromate-graphy/mass spectrometry analysis

**Peak**	**Compound**	**Percentage**
1	Methyl salicylate	0.11
2	Chavicol	0.14
3	Eugenol	85.04
4	Trans-Caryophyllene	1.86
5	Beta-Selinene	0.26
6	Acetoeugenol	12.02
7	Caryophtllene oxise	0.24
8	Adamantane	0.10
9	Dehydrodieugenol	0.21

All kids used in this study were horned, because their right horns gradually were growing up during the study.

**Fig. 1:**
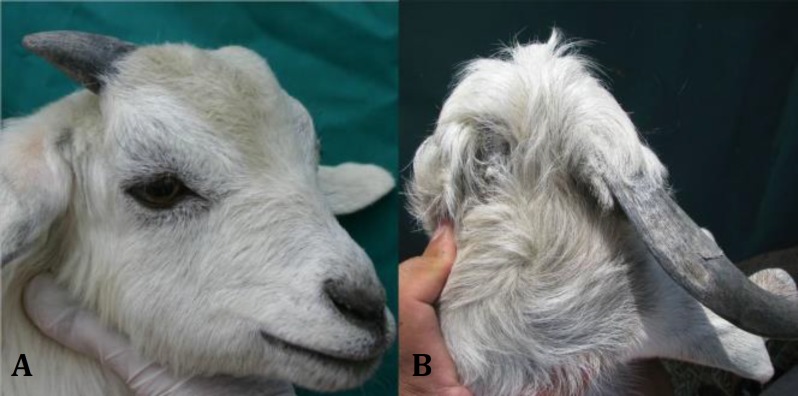
Horn growth arrest in the left side; **A)** two months and **B)** 12 months after injection of clove oil essence in the horn bud

**Fig. 2:**
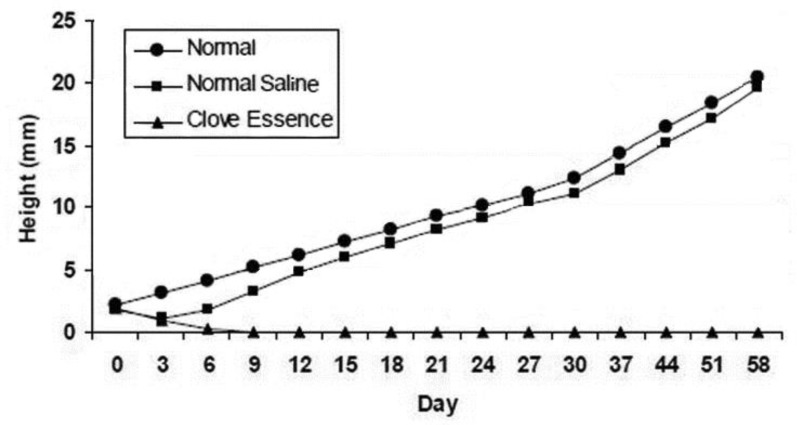
The growth rates of the horn in the right side (Normal), and in the left horn bud after injection of normal saline and clove essence in the kids, during the 2-month study

Results of the current study showed that the clove essence completely stopped horn growth in all kids of group 1 (Figs. 1A and 1B), whereas horn growth was observed in left horn of group 4 and there were no significant differences in horn growth rate between left and right horns in this group (Fig. 2).

We did not observe any noticeable changes in horn bud area following injection of normal saline in animals of groups 4, but injection of clove essence in group 1 induced significant changes in horn bud skin region including swelling and redness after 48 hr, then gradually skin necrosis was occurred during week 1 after injection.

**Fig. 3:**
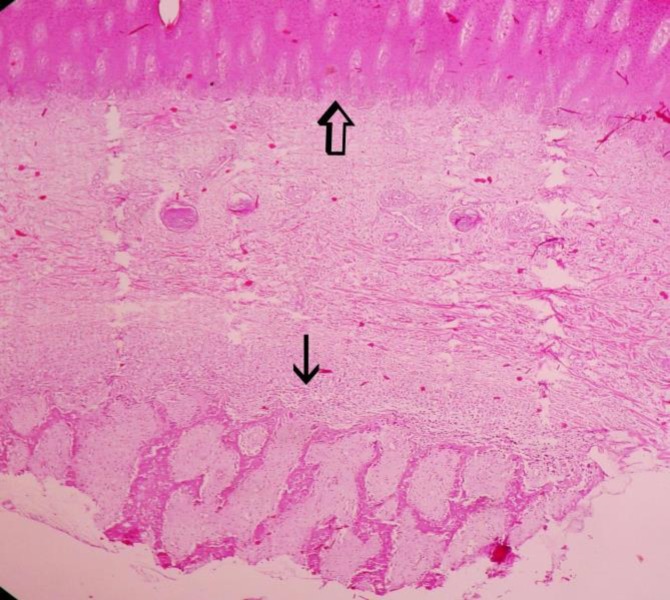
The normal horn bud as a cornified epithelium (open arrow) attached to the bone (thin arrow), (H & E, 40×).

**Fig. 4:**
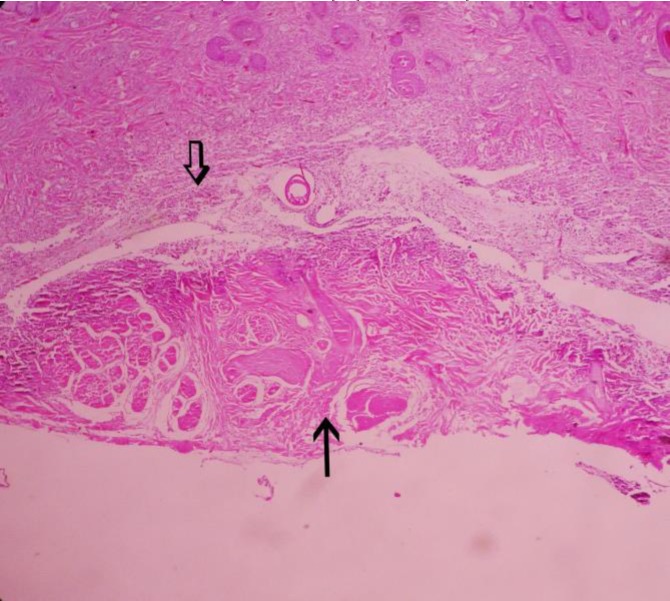
Complete necrosis of the horn bud (thin arrow) associated with mild infiltration of neutrophils (open arrow) after 5 days of clove oil essence injection, (H & E, 100×).

The microscopic study of right horns in groups 2 and 3 showed the normal horn bud as a cornified epithelium attached to the bone (Fig. 3). In the left horn of group 2, five days after injection of clove essence, complete necrosis of horn bud with a few neutrophils infiltrated around the necrotic tissues was observed. The epidermis, over the horn buds showed necrosis and ulceration. There was a mild infiltration of neutrophils in the surface of necrotic epidermis. Necrotic tissues and degenerated neutrophils formed scab on the ulcerated area. Underlying dermal tissues revealed necrosis and collagenolysis (Fig. 4).

**Fig. 5.**
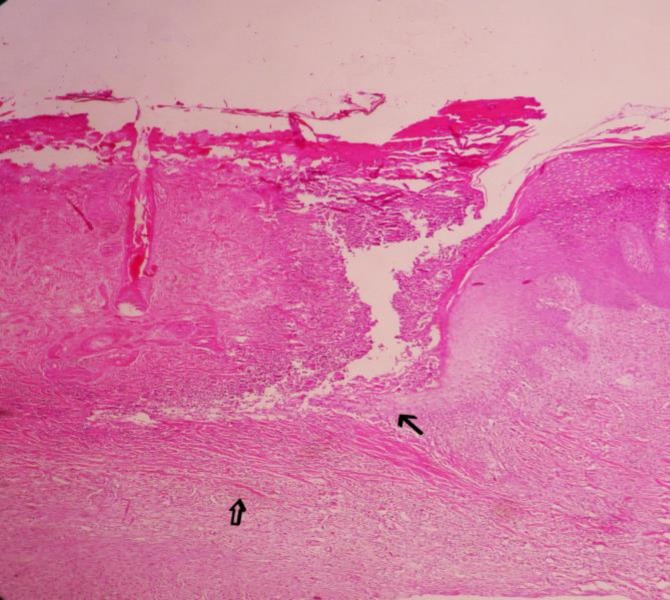
Re-epithelialization of epidermis (thin arrow) and developing of granulation tissues in the dermis after 10 days of clove oil essence injection in horn bud (open arrow), (H & E, 40×).

In the left horn of group 3, 10 days after injection of clove essence in horn bud, re-epithelialization started from around the necrotic area and grew under scab and granulation tissues formed in the dermis and healing processes developed (Fig. 5). 

## Discussion

Dehorning adult goats is not as easy as dehorning cattle. Because of serious complications following dehorning in adult goat, they are ideally disbudded as infants.^23^ The prolifrative area at the base of the horn is very much more extensive in young goats than in calves. Thus for successful disbudding a larger area needs to be destroyed and the dehorning of adult goat exposes the very large frontal sinuses at the base of the horn.^24^ Removal of large horns creates an opening into the frontal sinus that requires bandaging until the wound heals by second intention. Bandage maintenance can be technically difficult and cumbersome for the owners. Sinusitis, miasis, and prolonged healing are complications that may occur when using an open technique. ^7^^,^^23^

Disbudding of goat kids is a problematic procedure. One potential complication of disbudding is abnormal horn regrowth, or scar formation, which must be removed by other dehorning techniques.^6^

The horn buds grow extremely rapidly in goats and for satisfactory results disbudding should be performed when the kid is two to seven days old.^24^ Early disbudding of horns in the first few days of life with a hot iron is common, and the preferred practice in goat herds. If goats are not disbudded at an early age and have large horns, hot iron horn removing techniques are not amendable.^6^ Thermal disbudding in goat kids induces acute pain and stress, high intensity behaviours, and acute cortisol increase. So it is necessary to use anaesthesia/analgesia to avoid pain and stress before administration of disbudding methods.^25^ Relatively profound sedation or local analgesia can be achieved before dehorning performance. Because of the smaller size of most goats in comparison with other ruminants, and their high sensitivity to local anesthetics and anesthesia medications, it is important to consider the possibility of toxicity when applying analgesia/anesthesia.^26^ Meningoencephalitis caused by thermal disbudding is one of the tragic diseases in goat kids. Anatomically, the frontal bone of kids, unlike that of calves, is thin and horn buds is relatively large and the frontal sinus is undeveloped. Thermal damage to underlying bone, meninges and brain can results from prolonged or excessive pressure with the hot iron.^9^^,^^27^

Caustic paste disbudding is caused by the chemical burn of underlying tissue. Care has to be taken to prevent paste running onto face and eyes. Caustic paste disbudding causes intense pain from the first minutes after paste application and some behavioral signs of distress still remain at 3 hr after the procedure. The caustic paste is notorious for causing chemical burns on other parts of the goat or on his/her pen mates. To use caustic paste, the farmer must make sure that the kid is kept apart from others, so that it doesn’t rub the chemical on the udder of its mother or the faces of its friends and that it is kept out of the rain so that rain water doesn’t wash the chemical into the goat’s eyes.^28^

Eugenol is the main component of oil of cloves. It is used as a fragrance and flavoring agent, an insect attractant, and as a topical antiseptic and anti-inflammatory analgesic in dentistry.^18^ *Eugenia caryophyllata* has been used in traditional public medicine to relieve nasal obstruction and musculoskeletal pain which imply anti-inflammatory activity for the plant.^29^ Analgesic, anesthetic, spasmolytic and antibacterial effects of EC have been demonstrated by several scientific studies.^29^^,^^30^

Despite the extensive clinical use, eugenol is cytotoxic to various cell types though its mechanism of cytotoxicity has been unknown. Reportedly, eugenol reduced the growth of cells *in vitro*.^18^ Anpo *et al*. suggested that cytotoxic effect of eugenol can be associated with oxidative DNA damage by its metabilites. They also suggested that eugenol-related compound may act like non-steroidal anti-inflammatory drugs.^18^ In several studies, it has been stated that eugenol can inhibit neoplastic cell growth. Okada *et al*. demonstrated that eugenol induced apoptosis in oral tumor cells.^19^ Gosh *et al.* showed that eugenol could inhibit melanoma growth by inducing apoptosis and cell proliferation arrest.^20^ Their results showed eugenol is a potent inhibitor growth of melanoma cells, causes significant tumor growth delay, decreases size of tumor and inhibits melanoma invasion and metastasis. Eugenol arrests cells in the S phase of cell cycle, and induces apoptosis, and is not mutagenic.^20^ It has been shown eugenol is effective to inhibit papillomas.^31^

In our study horn growth arrest following injection of clove oil essence in horn bud can be attributed to different mechanisms. It seems that existence of eugenol as a main component of clove oil can inhibit proliferation in horn germination cells and induce apoptosis in these cells. Also eugenol may have cytotoxic effect on cells in horn bud and consequently induce cell necrosis, as the histopathological examination in the present study confirmed it. Koger described a suitable and safe method for dehorning in calves including injection of calcium chloride as a necrotizing agent in horn bud.^32^ An overall success rate of 70% has been achieved in his study. The calves were sedated before injection and injection site were prepared aseptically. He stated that without tranquilization or anesthesia, injection of the necrotizing calcium solutions caused objectionable pain and strong physical restrain was necessary. In the current study, because of anti-inflammatory and analgesic effect of eugenol,^18^ we did not use local anesthetics and/or sedative drugs before injection and the animals were only physically restrained at the time of injection. 

According to the results of this study injection of clove essence can be an effective method to stop horn growth without any undesirable effects on clinical parameters. This technique is easy for the operator and less stressful for the kids.
